# *Rigidoporus corticola* Colonization and Invasive Fungal Disease in Immunocompromised Patients, United States

**DOI:** 10.3201/eid2804.211987

**Published:** 2022-04

**Authors:** Alvaro C. Laga, Jessica W. Crothers, Connie F. Cañete-Gibas, Nathan P. Wiederhold, Isaac H. Solomon

**Affiliations:** Brigham and Women’s Hospital and Harvard Medical School, Boston, Massachusetts, USA (A.C. Laga, I.H. Solomon);; University of Vermont Medical Center, Burlington, Vermont, USA (J.W. Crothers);; University of Texas Health San Antonio, San Antonio, Texas, USA (C.F. Cañete-Gibas, N.P. Wiederhold)

**Keywords:** fungi, respiratory infections, *Rigidoporus*, *Oxyporus*, *corticola*, filamentous basidiomycetes, opportunistic fungal infection, pathology, emerging fungal disease, United States

## Abstract

We report 2 cases of *Rigidoporus corticola* (*Oxyporus corticola*) infection in humans in the United States. Clinical manifestations consisted of angioinvasive fungal sinusitis in 1 patient and pulmonary intracavitary fungus ball in the other patient. These cases illustrate previously undescribed clinicopathologic manifestations of infection by this filamentous basidiomycete in humans.

*Rigidoporus corticola* is a white-rot decay fungus of plants and is widely distributed in North America and Europe (*1*). *R*. *corticola* is a filamentous basidiomycete in the *Agaricomycotina* subphylum, also known as *Oxyporus corticola* because no morphologic differences or type species are in the same phylogenetic clade. *Rigidoporus* is the preferred name because it was described prior to *Oxyporus* (*2*,*3*). 

Filamentous basidiomycetes occur in the environment as gilled mushrooms and shelf or bracket fungi (*4*). Filamentous basidiomycetes (other than *R. corticola*) have been documented in immunocompetent and immunocompromised persons (*5*,*6*), but *R. corticola* has not been established as an agent of human disease. We describe the clinical, microbiologic, and histopathologic features of 2 cases of human *R. corticola* infection in the United States (Table).

**Table:** Clinical, microbiologic, and histopathologic features of *Rigidoporus corticola* infection in 2 immunocompromised patients, United States*

Characteristics	Patient 1	Patient 2
Age, y/sex	43/M	63/M
Underlying conditions	Acute myeloid leukemia, chronic granulomatous disease, hematopoietic stem cell transplant	Recurrent lung cancer, chemotherapy
Clinical manifestations	Hemifacial pain; sinusitis	Right lung nodule
Identification method	Broad-range fungal PCR, then sequencing from tissue culture for internal transcribed spacer and D1/D2 subregions (98.5%–100% identity)	Broad-range fungal PCR assay, then sequencing from formalin-fixed, paraffin-embedded tissue
Susceptibility testing	Posaconazole (MIC 8 µg/mL), voriconazole (MIC 0.5 µg/mL), isavuconazole (MIC 1.0 µg/mL)	Not done
Pathologic diagnosis	Invasive fungal sinusitis	Fungus ball
Specimen type	Sinus debridement tissue	Lung wedge resection
Histologic pattern	Hyphae invading vessels and bone	Hyphae in sclerotic cavity; no invasion
Branching degree	Predominantly 90°	Predominantly 90°
Clamp connections	Y	N
Vascular invasion	Y	N
Treatment	Surgical debridement, amphotericin B, voriconazole, isavuconazole, olorofim	Surgical debridement
Response	No disease on endoscopy at 6 mo; new pulmonary nodule at 9 mo	No disease after resection
Outcome	Death from invasive fungal disease aftet 10 mo	Death from recurrent lung cancer after 11 mo

## The Study

**Figure 1:**
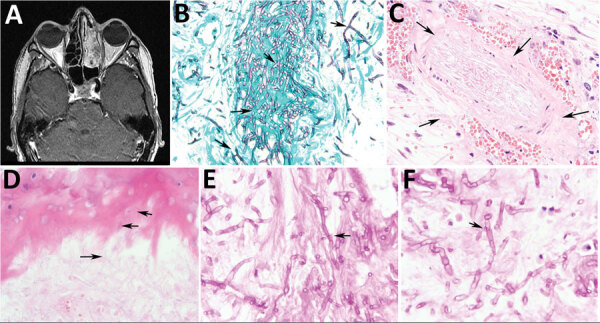
Radiologic and pathologic findings in a 43-year-old immunocompromised man with chronic granulomatous disease and diagnosed invasive fungal sinusitis caused by *Rigidoporus corticola* (*Oxyporus corticola*) infection, United States. A) Magnetic resonance imaging of the head showing mucosal thickening and near opacification in the left frontal, left ethmoid, left maxillary, and left sphenoid sinuses, and the left nasal cavity. No findings suggest extra-sinus extension. B) Debrided sinus tissue stained with Gomori methenamine silver. Arrows indicate numerous septate hyphae with predominantly right-angle branching. Original magnification ×600. C) Hematoxylin and eosin stain of blood vessels. Arrows indicate permeation (angioinvasion). Original magnification ×600. D) Hematoxylin and eosin stain of the sinonasal ossicles. Arrows indicate infiltration consistent with invasive fungal disease. Original magnification ×600. E, F) Periodic acid–Schiff-diastase–stained sinus tissue. Arrows indicate bulge-like (E) and hook-like (F) hyphal outgrowths potentially representing clamp connections_,_ characteristic of filamentous basidiomycetes, that were detected upon careful examination. Original magnification ×1,000.

The first patient was a 43-year-old man with chronic granulomatous disease and recurrent acute myeloid leukemia in whom hemifacial pain developed after he received induction chemotherapy. Magnetic resonance imaging showed mucosal thickening and opacification of the patient’s left sinuses (Figure 1, panel A). Nasal endoscopy revealed a black eschar on the middle turbinate, which our clinical colleagues interpreted as highly characteristic of mucormycosis, and we performed surgical debridement. Histologic sections showed numerous thin hyaline septate hyphae (≈5 µm in diameter) with predominantly 90°-angle branching but no conidiogenous cells or conidia (Figure 1, panel B). We noted vascular invasion and infiltrative growth into sinonasal ossicles (Figure 1, panels C, D). Careful examination revealed bulge and hook-like hyphal outgrowths compatible with clamp connections (Figure 1, panels E, F), which are characteristic of filamentous basidiomycetes. Concurrent fungal culture on Sabouraud dextrose agar grew white cottony colonies. Lactophenol cotton blue preparation from subcultures onto potato flake agar revealed thin sterile hyphae. We sent a culture to the Fungus Testing Laboratory (FTL) at the University of Texas Health Science Center (San Antonio, Texas, USA). FTL also noted growth on benomyl agar suggesting a possible basidiomycete. FTL amplified genomic DNA from the isolate and sequenced for the internal transcribed spacer (ITS) and large subregion (D1/D2) of the 28S rRNA gene (BMBC-R/NL4). Compared with sequences from the NCBI database (https://www.ncbi.nlm.nih.gov), most blastn matches were *R. corticola*, 98.53%–100% similarity for ITS and 98.55%–100% for D1/D2; subsequent phylogenetic analysis confirmed *R. corticola* (Appendix). Antifungal susceptibility testing demonstrated similar MIC values for voriconazole (0.5 μg/mL) and isavuconazole (1 μg/mL) but a high MIC (8 μg/mL) for posaconazole. The patient was initially treated empirically with liposomal amphotericin B for 20 days, then was transitioned to voriconazole after fungal identification and susceptibility testing. However, the patient experienced recurrent episodes of neutropenic fever and died 10 months later of invasive fungal disease. We presumed the infection to be aspergillosis on the basis of increased serum galactomannan on sequential samples, but this assumption was not confirmed by culture or PCR.

**Figure 2:**
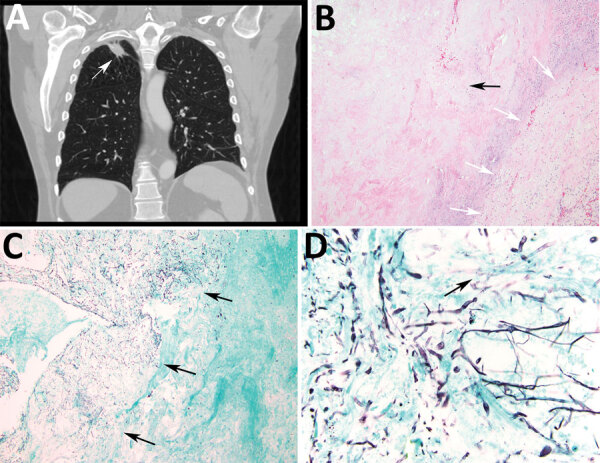
Radiologic and pathologic findings in a 61-year-old immunocompromised man with a history of lung adenocarcinoma and a new 2.4 cm right upper lobe mass determined to be a pulmonary fungus ball caused by *Rigidoporus corticola* (*Oxyporus corticola*) infection, United States. A) Chest computed tomography scan. Arrow indicates a 2.4 × 2.2 cm ovoid pulmonary mass in the right upper lobe. B) Hematoxylin and eosin–stained histologic sections of the resected mass. Black arrow indicates a cavitary lesion with a necrotic center. White arrows indicate peripheral fibrous capsule. Original magnification ×100. C, D) Gomori methenamine silver–stained histologic sections. C) Arrows indicate numerous hyphae within the cavity, but no evident invasion into blood vessels or surrounding tissue. Original magnification ×40. D) Arrow indicates septate thin hyphae evident in the center of the cavity. Original magnification ×600.

The second patient was a 63-year-old man with a history of lung adenocarcinoma and a new 2.4 cm right upper lobe mass resected for possible recurrence (Figure 2, panel A). Histologic sections showed a cavitary lesion with necrotic center and peripheral fibrous capsule (Figure 2, panel B). Sections stained with Gomori methenamine silver showed numerous septate hyphae in the cavity but no invasion into blood vessels or surrounding tissue (Figures 2, panels C, D). Neither conidiogenous cells nor conidia were evident; we did not identify clamp connections in samples after careful examination. The Centers for Disease Control and Prevention (Atlanta, Georgia, USA) identified *O. corticola* (*R. corticola*) by PCR from formalin-fixed, paraffin-embedded (FFPE) tissue sections of the wedge resection. We did not attempt culture, and the patient did not receive antifungal therapy. He died 11 months later of recurrent lung adenocarcinoma.

## Conclusions

Filamentous basidiomycetes are common environmental fungi increasingly recognized as agents of human disease, causing saprobic colonization of the respiratory tract, allergic disease, and invasive fungal infections (*6*,*7*). A review of 218 pathogenic cases reported worldwide found the most common species are *Schizophylum commune* (52%), *Hormographiella aspergillata* (5.9%), and *Ceriporia lacerata* (5%) (*7*). Anecdotal reports describe galactomannan and β-d glucan production by filamentous basidiomycetes such as *H. aspergillata*, suggesting that testing for these polysaccharides might help diagnose invasive disease. 

Two cases of *R. corticola* infection have been reported in canines. One was a disseminated *R. corticola* infection reported in 2009 in a German shepherd dog, which was ultimately euthanized 20 months after clinical presentation because of progressive disease and central nervous system involvement despite therapy with itraconazole and terbinafine (*1*). Another case, in 2012, was reported as generalized lymphadenopathy in a beagle, which was confirmed by sequencing of a culture isolate to be caused by *R. corticola* (*8*). This dog also was euthanized because of progressive disease despite empiric treatment with itraconazole 14 weeks after clinical presentation (*8*). Histopathology from both canine cases showed septate branching hyphae with parallel to slightly tapering walls.

We document *R. corticola* as an agent of human disease in 2 immunocompromised patients; the fungus colonized the pulmonary cavity of one patient and caused invasive sinusitis in the other. The spectrum of disease we describe resembles that observed for other filamentous basidiomycete fungi. Our findings align with the literature; these organisms are difficult to identify by in vitro culture because of lack of sporulation and are not yet reliably identifiable by mass spectrometry (*9*). Standard sequencing of 18S, 28S, or ITS rRNA and other fungal genomic regions can assist in species-level identification, but accurate identification is limited by available sequencing databases (*10*–*12*). However, molecular identification of fungi by itself is insufficient to establish pathogenicity because it might not distinguish between an agent of disease and a contaminant, particularly in cases involving common environmental fungi and uncommon species not established as agents of human disease, such as *R. corticola*. The 2 clinical cases we describe were detected by histopathology, confirming a pathogenic role in invasive fungal disease in 1 case and colonization of an existing cavity in the other, according to current guidelines (*13*); fungal sequencing of a culture isolate and FFPE tissue subsequently confirmed identification. These data support observations that filamentous basidiomycetes are being recognized more frequently in clinical specimens and that identification by fungal cultures and biochemical methods remains challenging. Consequently, filamentous basidiomycetes and their burden of disease likely are underrecognized. 

Although we performed susceptibility testing against 1 isolate according to the Clinical and Laboratory Standards Institute M38 reference standard (*10*), no breakpoints for antifungal agents against this species have been established. Of note, the MIC for posaconazole was elevated compared with other basidiomycetes reported to cause human infections, such as *S. commune*. Although data are limited, of 5 *R. corticola* isolates in the FTL database with MIC results for posaconazole, 4 had elevated posaconazole MICs (range 2.0 to >16 μg/mL), but voriconazole MICs were lower (range 0.25–1.0 μg/mL). Lack of evidence-based treatment guidelines might have contributed to illness in these patients, both of whom died from complications of their underlying diseases <1 year after seeking care for *R. corticola* infection. 

Although filamentous fungi cannot be speciated by histology, detection of the characteristic clamp connections should prompt consideration of filamentous basidiomycetes in the differential diagnosis. Admittedly, this finding might be rare because most clinical isolates are reportedly monokaryons (*14*), but 1 study documented clamp connections in 78% of clinical isolates of *S. commune* and *C. lacerata* (*15*). 

In summary, our report adds *R. corticola* to the list of increasingly recognized filamentous basidiomycete fungi associated with human disease. Clinicians should consider *R. corticola* when assessing immunocompromised patients for invasive fungal infections or respiratory tract colonization.

Appendix Additional information on *Rigidoporus corticola* colonization and invasive fungal disease in immunocompromised patients, United States.
