# Supplementary material for: Rigidoporus corticola Colonization and Invasive Fungal Disease in Immunocompromised Patients, United States
Source: Emerg Infect Dis. 2022 Apr;28(4):856–9. doi: 10.3201/eid2804.211987 (PMC8962884; doi:10.3201/eid2804.211987)
Supplement: Appendix — Additional information on Rigidoporus corticola colonization and invasive fungal disease in immunocompromised patients, United States. [file 21-1987-Techapp-s1.pdf]

Article DOI: https://doi.org/10.3201/eid2804.211987

Rigidoporus corticola Colonization and Invasive Fungal Disease in Immunocompromised Patients, United States

Appendix

Appendix Table. GenBank accession numbers of internal transcribed spacer (ITS) and large subunit (LSU) sequences of representative taxa listed in Wu et al. reference, and *Rigidoporus coricola* sequences generated from the collection of the Fungus Testing Laboratory (FTL) at the University of Texas Health San Antonio, Texas, USA*

FTL accession no.	ITS GenBank accession no.	LSU GenBank accession no.
UTHSCSA DI22–1	OM127879	OM128160
UTHSCSA DI22–2	OM127880	OM128161
UTHSCSA DI22–3	OM127881	OM128162
UTHSCSA DI22–4	OM127882	OM128163
**UTHSCSA DI22–5**	OM127883	OM128164
UTHSCSA DI22–6	OM127884	OM128165
UTHSCSA DI22–8	OM127885	OM128166
UTHSCSA DI22–9	OM127886	OM128167
UTHSCSA DI22–10	OM127887	OM128168
UTHSCSA DI22–11	OM127888	OM128169
UTHSCSA DI22–12	OM127889	OM128169
UTHSCSA DI20–342	MW476707	MW478302

*(*1*) Wu et al. 2017. Bold text indicates case 1 isolate.

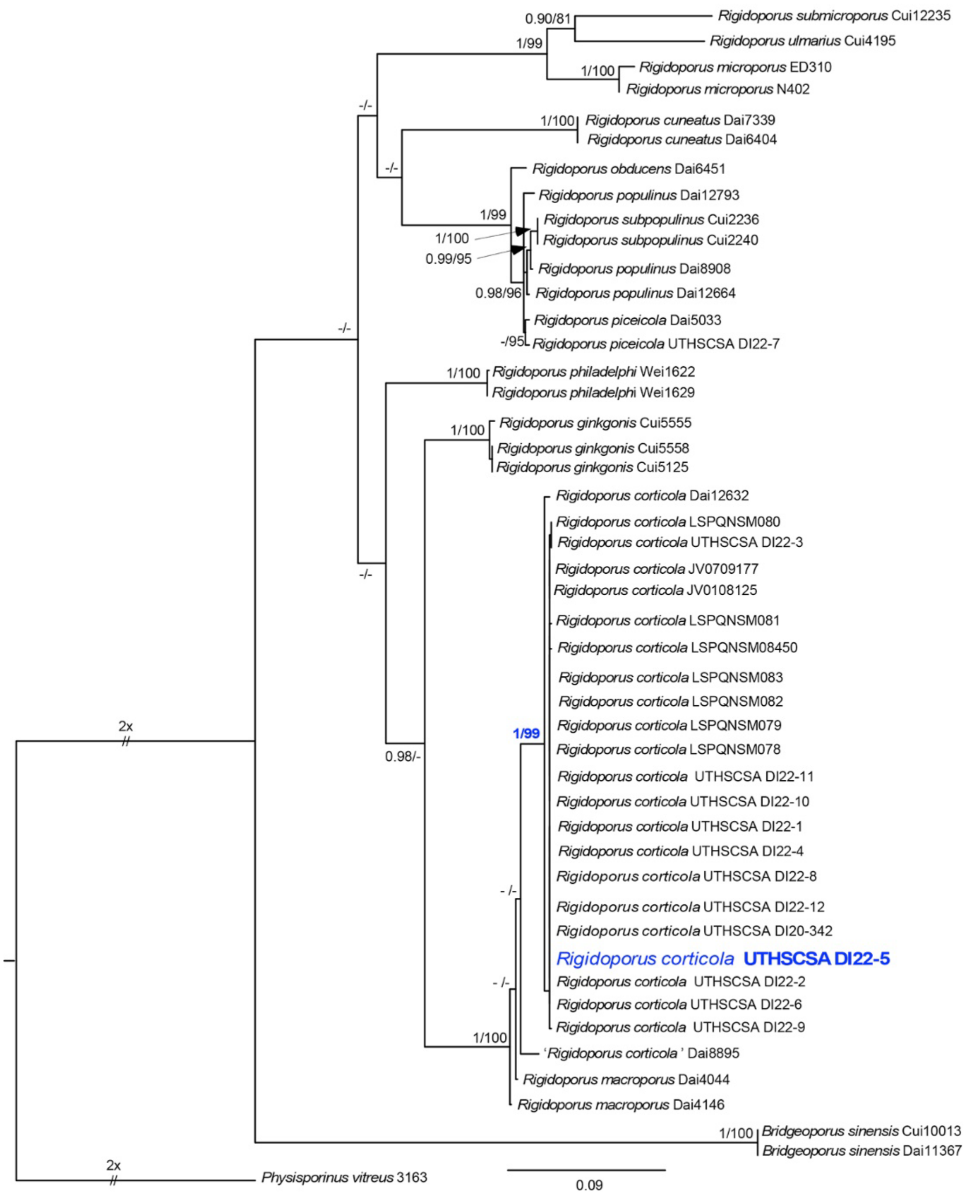

Appendix Figure. Phylogenetic tree of internal transcribed spacer (ITS) and large subunit (LSU) sequences of representative taxa listed in Wu et al. (*1*), and *Rigidoporus coricola* sequences generated from the collection of the Fungus Testing Laboratory (FTL) at the University of Texas Health San Antonio, Texas, USA. The tree shows a subset of the backbone for *Rigidoporus* and related genera and the relationship of isolate UTHSCSA DI22–5 (blue text) from case-patient 1 with other FTL isolates and representative species of *Rigidoporus*. The tree was inferred from combined ITS and partial LSU (D1/D2) sequences by using maximum likelihood general time-reversible plus gamma distribution plus invariable site substitution model in IQ-TREE version 2.1.3 (http://www.iqtree.org). Bayesian posterior probabilities were calculated by using MrBayes version 3.2 (http://nbisweden.github.io/MrBayes); values $\geq$0.95 and bootstrap values $\geq$80% are shown at the nodes. Scale bar indicated nucleotide substitutions per site. UTHSCSA DI, University of Texas Health at San Antonio deidentified number.

Reference

1. Wu F, Chen JJ, Ji XH, Vlasák J, Dai YC. Phylogeny and diversity of the morphologically similar polypore genera *Rigidoporus*, *Physisporinus*, *Oxyporus*, and *Leucophellinus*. Mycologia. 2017;109:749–65. PubMed https://doi.org/10.1080/00275514.2017.1405215
